# Computing Power and Sample Size for Informational Odds Ratio ^†^

**DOI:** 10.3390/ijerph10105239

**Published:** 2013-10-21

**Authors:** Jimmy T. Efird

**Affiliations:** Center for Health Disparities Research and Department of Public Health, Brody School of Medicine, Greenville, NC 27858, USA; E-Mail: jimmy.efird@stanfordalumni.org; Tel.: +1-650-248-8282

**Keywords:** informational odds ratios, power, sample size

## Abstract

The informational odds ratio (IOR) measures the post-exposure odds divided by the pre-exposure odds (*i.e.*, information gained after knowing exposure status). A desirable property of an adjusted ratio estimate is collapsibility, wherein the combined crude ratio will not change after adjusting for a variable that is not a confounder. Adjusted traditional odds ratios (TORs) are not collapsible. In contrast, Mantel-Haenszel adjusted IORs, analogous to relative risks (RRs) generally are collapsible. IORs are a useful measure of disease association in case-referent studies, especially when the disease is common in the exposed and/or unexposed groups. This paper outlines how to compute power and sample size in the simple case of unadjusted IORs.

## 1. Introduction

A useful measure of association is the informational odds ratio (IOR) [1]. In the unadjusted case, the IOR is computed as (a/b)/(g/h), where a = number of exposed diseased individuals, b = number of exposed non-diseased individuals, g = number of diseased individuals, and h = number of non-diseased individuals (Table 1, Equation (1)). The IOR is equivalent to the post-exposure odds divided by the pre-exposure odds and is interpreted as an outcome measure of information gained after knowing exposure status (Equation (2)). The measure resembles the traditional odds ratio (TOR) (*i.e.*, TOR = (a/b)/(c/d), where c = number of non-exposed disease individuals, d = number of non-exposed non-disease individuals), except that the probability terms in the denominator are not conditioned on the absence of exposure. 

A key advantage of IORs is that the Mantel-Haenszel adjusted ratio estimates are collapsible (*i.e.*, the combined crude ratio will not change after adjusting for a variable that is not a confounder) [1]. In contrast, adjusted TORs are not collapsible. IORs also are a useful and meaningful measure of association in case-reference studies of common diseases (e.g., obesity, diabetes) because their practical interpretation does not depend on estimating relative risk (RR) (*i.e.*, rare disease assumption not required). 

Prior to conducting a study it is important to determine how large a sample is needed to be reasonable confident that estimates are precise and suitable for answering *a priori* hypotheses. Alternatively, one may specify the sample size and then compute the study power required to reject the null hypothesis given that it is false. This paper presents simple formulas for computing power and sample size for IOR. 

**Table 1:** A 2 × 2 contingency table.

Disease → ↓Exposure	D	D	Total
E	a = 2,352	b = 1,600	e = 3,952
E	c = 912	d = 1,600	f = 2,512
Total	g = 3,264	h = 3,200	i = 6,464

The IOR is computed from the above 2 × 2 contingency table as:

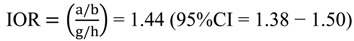
(1)
where the 95%CI is based on the robust variance estimate for log (IOR) [1]. The equivalence between IOR and the post-exposure odds divided by the pre-exposure odds is shown below:

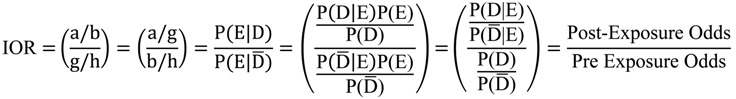
(2)

## 2. Methods

There exist two types of error in classical statistical hypothesis testing [2]. In the current context, a rejection error (also known as a “Type I” or “α” error) occurs when the null hypothesis (*H_0_*:IOR = 1) *versus* the alternative hypothesis (*H_A_*:IOR ≠ 1) is falsely rejected, *i.e.*, α = P(reject *H_0_* | *H_0_* is true). An acceptance error (also known as a “Type II” or “β” error) occurs when the null hypothesis is falsely accepted, *i.e.*, β = P(do not reject *H_0_* | *H_0_* is false). The “power” of the test of hypothesis is defined as (1 − β) and denotes the probability of correctly rejecting the null hypothesis, *i.e.*, P(reject *H_0_* | *H_0 _* is false). The power also conveys the likelihood that a particular research design will detect a deviation from the null hypothesis given that one exists. Another important concept in experimental design and hypothesis testing is the “sample size” of a test. Sample size denotes the number (n) of experimental units (e.g., people, animals, widgets) needed to achieve a specified power at the α-level of statistical significance. Several factors influence the sample size including Type I and Type II error, and the underlying variability of the sampling distribution. 

Power and sample size for IORs may be computed by a simple rearrangement of the general formulas for marginal risk ratios [3]. Letting p_1_ = proportion of diseased individual who are exposed, p_0_ = proportion of non-diseased individuals who are exposed, r = ratio of non-diseased to diseased individuals, z_α/2_ = 100(1 − α/2) centile of the standard normal distribution, Z_β_ = the standard normal deviate corresponding to β = (1 − power), it follows that Z_β_ = [n·(p_1_ − p_0_)^2^·r/(r + 1)·ξ·(1 − ξ)]^1/2^ − Z_α/2_ and n = (Z_α/2_ + Z_β_)^2^·ξ·(1 − ξ)·(r + 1)/(p_1_ − p_0_)^2^·r, where ξ = (p_1_ + r·p_0_)/(1 + r), and p_1_ = p_0_·IOR. Power then equals the probability that an observation from the standard normal distribution is less than or equal to Z_β_. The above formulas assume a log-normal distribution for IOR and the use of a robust variance estimate for the logarithm of IOR based on the delta method [1,3]. Many commonly available statistical packages provide routines for computing power and sample size for hypothesis tests involving unadjusted RRs. These routines may be adapted to compute power and sample size for IORs by transposing the input data matrix. Results from the examples below may be used to confirm that the input matrix was properly transposed. Slight differences in the results may be due to variations in the underlying sampling distribution, algorithms and/or numerical methods used by a particular statistical package. Under general admissibility conditions, computations will converge in distribution and yield asymptotically equivalent results [4]. 

## 3. Examples

**Figure 1:**
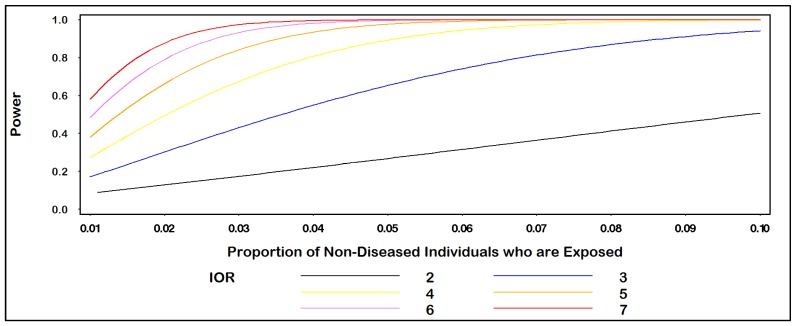
Power for IOR by proportion of non-diseased individuals who are exposed.

**Figure 2:**
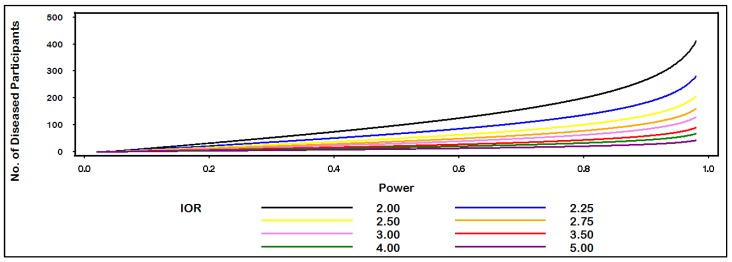
Sample size for IOR by power.

Assuming an equal number of diseased (n_1_ = 100) and non-diseased (n_0_ = 100) individuals, the plot in Figure 1 gives power for IOR = (2, 3, 4, 5, 6, 7) for values of p_0_ ranging from 0.01 to 0.10. For example, when p_0_ = 0.04, the power to detect an IOR of at least 4.0 equals 80.7% at the α = 0.05 level of statistical significance. In Figure 2, we see that 200 diseased (and 200 non-diseased) individuals are needed to be sufficiently powered (>80%) to detect an IOR of at least 2.0 at the α = 0.05 level of statistical significance when the proportion of non-diseased individuals who are exposed equals 0.10. 

(Alpha = 0.05; No. non-diseased Pts. = 100; non-diseased: diseased ratio = 1.0).

(Alpha = 0.05; proportion of non-diseased who are exposed = 0.10; non-diseased: diseased ratio = 1.0).

## 4. Discussion and Conclusions

An important property of adjusted IORs is their collapsibility and interpretability as an outcome measure of information gained after knowing exposure status (*i.e.*, post-exposure odds divided by the pre-exposure odds). While TORs approximate RRs and collapsibility under the rare disease assumption, they lack this property in the case of retrospective studies of a common disease. The distinction of IORs *versus* RRs is that the former may be used in case-referent studies. This is because IORs do not depend on exposure (risk) margins but rather on column disease margins. However, because IORs still are a marginal estimate similar to RR, both estimates share the property of collapsibility.

Based on the mirror relationship of IORs and RRs as marginal measures of association, the formulas used to compute power and sample size for RRs may be simply rearranged and applied to IORs. This is a particularly useful feature in practice given the availability of software for computing the power and sample size of RR estimates. 

The power and sample size formulas for IOR are based on asymptotic statistics and only should be used when the sample size is reasonably large and the sampling distribution for log (IOR) is approximately Gaussian. Similar to RRs, IORs are upwardly biased and actual power may be lower than the estimated one, at least for small sample sizes. Furthermore, the methods described for computing power and sample size apply to the simple case of unadjusted IORs and must be modified accordingly for more complex applications. 
